# Differential Effects of Biogas Slurry Topdressing on Winter Wheat (*Triticum aestivum* L.) Soil Enzyme–Microbe Interactions

**DOI:** 10.3390/microorganisms13112494

**Published:** 2025-10-30

**Authors:** Dongxue Yin, Baozhong Wang, Jiajun Qin, Wei Liu, Xiaoli Niu, Dongdong Chen, Jie Zhu, Fengshun Zhang

**Affiliations:** 1 College of Agricultural Equipment Engineering, Henan University of Science and Technology, Luoyang 471000, China; wbz666comeon@163.com (B.W.); qinj009@163.com (J.Q.); niuxiaoli88@126.com (X.N.); chenddxuexi@163.com (D.C.); zj1270316684@163.com (J.Z.); 16627732157@163.com (F.Z.); 2 College of Agriculture, Henan University of Science and Technology, Luoyang 471000, China; liuwei0502636@163.com

**Keywords:** biogas slurry, soil enzymes, microbial communities, enzyme and microbe correlation

## Abstract

This study explored how top-dressed biogas slurry at winter wheat’s (*Triticum aestivum* L.) jointing stage (JS) and grain-filling period (GP) affects soil enzyme–microbe interactions, aiming to address nutrient supply–crop demand mismatches. A field experiment with five treatments (water [CK], chemical fertilizer [CF], and three biogas slurry topdressing regimes [S1–S3]) was conducted. Soil samples (0–20 cm) were collected at JS, flowering stage (FS), GP, and reaping period (RP) to analyze soil properties (total nitrogen [TN], available phosphorus [AP], available potassium [AK], soil organic matter [SOM], ammonium nitrogen [AN], pH), enzyme activities (urease [UE], neutral phosphatase [NP], sucrase [SC], catalase [CAT]), and microbial community abundance (via Illumina NovaSeq sequencing). Results showed biogas slurry altered enzyme activities, microbial structure (e.g., Actinomycetota, Ascomycota), and their interactions by regulating soil properties. JS application boosted Pseudomonadota and UE activity, GP application increased Ascomycota and CAT activity, and S3 had the most complex enzyme–microbe network, enhancing nutrient cycling. The analysis indicated that UE activity was strongly and positively correlated with several bacterial phyla (e.g., Planctomycetota, Verrucomicrobiota) (*p* < 0.01) and fungal phyla (e.g., Ascomycota) (*p* < 0.01).

## 1. Introduction

Soil enzymes function as mediators and catalysts in soil biochemical reactions [1,2], whereas soil microbial communities act as principal drivers in sustaining soil ecological functions [3,4,5,6]. Soil enzymes are primarily secreted by soil microorganisms and are a direct reflection of microbial metabolic activity. Consequently, the structure and composition of the microbial community are the key drivers determining the soil enzyme pool and its activity. This causal relationship between the microbial community and enzyme activity is the fundamental engine driving carbon and nitrogen cycling, as well as nutrient transformation [7]. Their dynamic changes directly affect the sustainability of agricultural ecosystems. Lacava et al. [8] reported that the increased secretion and activity of enzymes such as phosphatases represent a critical adaptive response of certain microorganisms and plants to soil acidity and phosphorus deficiency. Rao et al. [9] found that β-glucosidase activity can serve as an important indicator for assessing soil health. Costa et al. [10] indicated that microbial-secreted polysaccharides and proteins can exert binding effects that tightly integrate soil particles, thereby forming stable soil aggregates. Li et al. [11] demonstrated that urease (UE) activity exhibits significant positive correlations with both soil organic carbon (SOC) and Actinobacteria abundance. This result suggests that SOC serves as a carbon and energy source for Actinobacteria, which secrete UE to facilitate nitrogen cycling processes. Therefore, elucidating the dynamic interactions between soil enzymes and microorganisms establishes a theoretical framework for examining the effects of biogas slurry application on winter wheat-cultivated soils.

Biogas slurry, an organic fertilizer rich in soil organic matter (SOM) and active microorganisms, achieves dual objectives of enhancing nutrient retention and increasing crop yield when applied to fields [12,13,14,15]. Several studies have shown that biogas slurry can significantly enhance the activities of various soil enzymes, including UE, phosphatase, and sucrase (SC) [16,17,18,19], thereby fostering a more favorable micro-ecological environment for microbial proliferation. Feng et al. [20] found in wheat studies that biogas slurry application alone increased catalase (CAT) activity by 12.3%, 19.1%, and 18.0% during the jointing, booting, and flowering stages (FS), respectively, compared to complete urea treatment. Yadav et al. [21] found that alkaline phosphatase exhibited higher activity than acid phosphatase, but the addition of biogas slurry balanced the activities of both, establishing a harmonized enzymatic profile. Abbas [22] found that the combined application of biochar, compost, and biogas slurry enhanced soil UE activity by up to 96% compared to the control. In addition to promoting soil enzyme activity, biogas slurry also markedly affects soil microbial community structure and function. Numerous studies have demonstrated that substituting chemical fertilizers with biogas slurry increases both the diversity and abundance of soil microbial communities, thereby enhancing the overall microbial environment [23,24,25]. For example, Abubaker et al. [26] found that applying cattle slurry to different soils significantly altered the bacterial community structure. Yu et al. [27] highlighted that high-concentration biogas slurry treatment results in approximately 50% greater relative abundance of pathotroph–symbiotroph fungi compared to the control. These findings indicate that biogas slurry application can remodel the enzyme–microbe interaction network in winter wheat-cultivated soils, with effects likely to show time-dependent differences. Atav et al. [28] discovered that in a one-year application of biogas slurry, the increase in CO_2_ respiration weakened after approximately 60 days, while the increase in microbial biomass lasted only 30 days. In a two-year application, the effect on CO_2_ respiration disappeared after 30 days, with no significant change in microbial biomass. Gryń et al. [29] found that the application of biogas slurry increased the total number of bacteria and actinomycetes in the soil, and to a lesser extent increased the total number of fungi.

Despite these advances, most research has focused on the static effects of biogas slurry application, with limited attention to systematic investigation of application timing. This knowledge gap has resulted in spatiotemporal mismatches between slurry recycling and crop nutrient requirements, restricting the synergistic functions of soil enzymes and microorganisms during critical periods of crop nutrient demand. Therefore, this study investigates the dynamic response patterns of winter wheat field soil properties (e.g., total nitrogen [TN], available phosphorus [AP], pH) to biogas slurry application at different growth stages based on the differential nutrient demands during key winter wheat growth periods (jointing stage [JS] and grain-filling period [GP]). Therefore, based on previous research, a scientific hypothesis is proposed: the regulatory effect of biogas slurry differs across key nutrient demand stages, and its application timing influences the microbial community structure and enzyme activity.

## 2. Materials and Methods

### 2.1. Experimental Design

**Figure 1:**
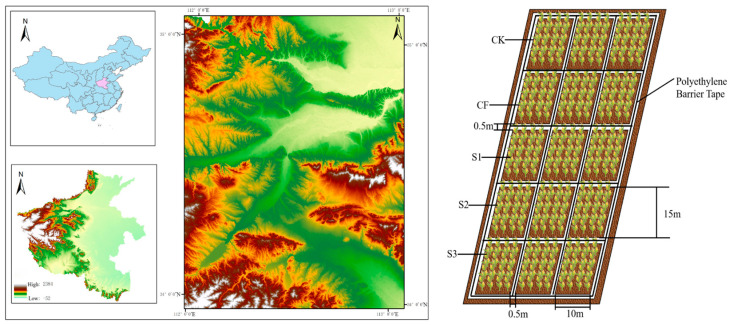
Experimental field location. CK: Topdressing with water at both JS and GP, CF: Topdressing with chemical fertilizer at both JS and GP, S1: Topdressing with biogas slurry at JS, followed by topdressing with chemical fertilizer at GP, S2: Topdressing with chemical fertilizer at JS, followed by topdressing with biogas slurry at GP, S3: Topdressing with biogas slurry at both JS and GP.

This study utilized winter wheat (*Triticum aestivum* L.) (‘Luohan 20′) as the experimental material at the Lankeshan Cooperative Farm in Xin’an County, Luoyang City, Henan Province, China, a region characterized by a temperate continental climate with mean annual temperature of 14.5 °C, approximately 223 frost-free days, annual precipitation of 578.2 mm (predominantly occurring from July to September), potential evaporation of 1200.0 mm, and sufficient solar radiation. The experimental soils were classified as clay loam with medium–high fertility. Initial soil chemical properties were as follows: pH 7.87, TN 1.24 g·kg^−1^, AP 31.33 mg·kg^−1^, available potassium (AK) 142.33 mg·kg^−1^, SOM 22.64 g·kg^−1^, and ammonium nitrogen (AN) 2.94 mg·kg^−1^. The specific location of the experimental field is shown in Figure 1.

This experiment comprised five treatments, each receiving a base application of 450 kg·ha^−1^ of chemical fertilizer. The specific topdressing regimens for each treatment during the JS and GP were as follows: (1) CK: Topdressing with 33.96 m^3^·ha^−1^ of water at both JS and GP; (2) CF: Topdressing with 150 kg·ha^−1^ of chemical fertilizer along with 33.96 m^3^·ha^−1^ of water at both JS and GP; (3) S1: Topdressing with a mixture of 16.98 m^3^·ha^−1^ of biogas slurry and 16.98 m^3^·ha^−1^ of water at JS, followed by topdressing with 150 kg·ha^−1^ of chemical fertilizer and 33.96 m^3^·ha^−1^ of water at GP; (4) S2: Topdressing with 150 kg·ha^−1^ of chemical fertilizer and 33.96 m^3^·ha^−1^ of water at JS, followed by topdressing with a mixture of 16.98 m^3^·ha^−1^ of biogas slurry and 16.98 m^3^·ha^−1^ of water at GP; (5) S3: Topdressing with a mixture of 16.98 m^3^·ha^−1^ of biogas slurry and 16.98 m^3^·ha^−1^ of water at both JS and GP. The plot for each treatment measured 30 m in length by 15 m in width. Each treatment was replicated three times. The biogas slurry used in this experiment was collected from normally operating digesters at Lankeshan Cooperative Farm in Xin’an County, Luoyang City, with the fermentation feedstock primarily consisting of mixed substrates including cattle manure, swine manure, and crop straws. The biogas slurry underwent 10-day sedimentation prior to application, followed by secondary filtration through a 100-mesh nylon sieve. Chemical analysis revealed the following composition: total solids 0.43%, volatile solids 34.92% (of total solids), AP 18.08 mg·L^−1^, AK 0.84 mg·L^−1^, TN 375.54 mg·L^−1^, and SOM 1.11 g·L^−1^.

### 2.2. Soil Sample Collection

Soil samples were collected from 0–20 cm depth using an auger and the five-point sampling method during the JS, flowering stage (FS), GP, and reaping period (RP) of winter wheat.

### 2.3. Soil Physicochemical Property Analysis

The soil physicochemical properties were determined using standardized analytical methods: (1) TN content via the Kjeldahl digestion method, (2) SOM content by potassium dichromate oxidation–ferrous sulfate back titration, (3) AP using the molybdenum-antimony anti-spectrophotometric method, (4) AK by flame emission spectrometry, (5) AN through potassium chloride extraction–continuous flow analysis, and (6) soil pH measured in a 1:2.5 soil-to-distilled water suspension; the value was recorded following the stabilization of the reading.

### 2.4. Determination of Microbial Community Relative Abundance

The soil metagenomic sequencing analysis was performed by Shanghai Majorbio Bio-pharm Technology Co., Ltd. (Shanghai, China) using the NovaSeq (Illumina, San Diego, CA, USA) for high-throughput sequencing. The raw data processing pipeline consisted of the following steps: (1) Quality control of raw reads was conducted using fastp 0.20.0, including precise identification and trimming of 3′ and 5′ adapter sequences, followed by filtering to remove low-quality reads (length < 50 bp, average base quality score (Q-value) < 20, or containing N bases), retaining high-quality paired-end and single-end sequencing data; (2) Valid data were assembled, and contigs with length ≥ 300 bp were selected as the final assembly results; (3) Using the SOAPaligner 2.21, high-quality reads from each sample were strictly aligned against a non-redundant gene database (similarity threshold set at 95%) to quantify the relative abundance of each gene across samples.

### 2.5. Determination of Soil Enzyme Activities

The four most representative enzymes were included in our study (Table 1). The activities of SC, CAT, UE, and NP were determined using assay kits (Solarbio Science and Technology Co., Ltd., Beijing Economic-Technological Development Area, Beijing, China) and measured with a Multiskan SkyHigh full-wavelength microplate reader (Thermo Fisher, Waltham, MA, USA) at the specific absorbance required for each enzyme. The CAT activity was determined by measuring the decomposition rate of H_2_O_2_ at 240 nm [30]. The reaction system contained 0.03 g of soil sample with specific buffer, and absorbance changes were measured after a 20 min incubation at 25 °C. The UE activity was assayed using the indophenol blue colorimetric method [31]. A total of 0.05 g of soil sample was incubated with urea substrate at 37 °C for 24 h, and the ammonia nitrogen product was detected at 630 nm. The SC activity was determined via the 3,5-dinitrosalicylic acid (DNS) method [32]. After incubating 0.03 g of soil with sucrose substrate at 37 °C for 24 h, the reducing sugars produced reacted with DNS reagent to form a reddish-brown amino compound, the absorbance of which was measured at 540 nm. The NP activity was determined using a phenol reagent-based microassay [33]. Specifically, 0.1 g of soil sample was incubated with disodium phenyl phosphate solution at 37 °C for 24 h. The S-NP-catalyzed enzymatic reaction yielded phenol and disodium hydrogen phosphate (Na_2_HPO_4_). Phenol concentration was quantified spectrophotometrically at 660 nm following its chromogenic reaction with 2,6-dibromobenzoquinone chlorimide under alkaline conditions.

### 2.6. Statistical Analysis

Microsoft Excel 2021 was employed to statistically organize the experimental data. Origin 2021 software was employed for conducting correlation analysis and plotting the changes in enzyme activity. The ‘tidyverse’ package in RStudio 3.5.3 was used for data processing and to generate stacked bar plots of microbial community relative abundance.

## 3. Results

### 3.1. Wheat Yield Response to Different Treatments

**Figure 2:**
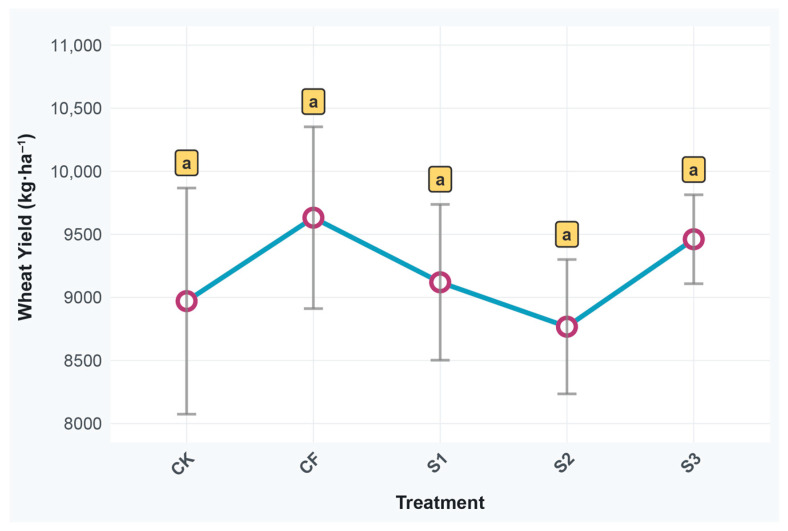
Relative abundance of bacterial communities in different treatments during various periods. CK: Topdressing with water at both JS and GP, CF: Topdressing with chemical fertilizer at both JS and GP, S1: Topdressing with biogas slurry at JS, followed by topdressing with chemical fertilizer at GP, S2: Topdressing with chemical fertilizer at JS, followed by topdressing with biogas slurry at GP, S3: Topdressing with biogas slurry at both JS and GP. The same letter a indicates no significant difference among treatments.

The yield data revealed that the CF treatment achieved the highest yield, with a value of 9632.00 kg·ha^−1^, followed by the S3 treatment at 9461.33 kg·ha^−1^ (Figure 2). In contrast, the yields of the S1, CK, and S2 treatments were 9120.00 kg·ha^−1^, 8970.67 kg·ha^−1^, and 8768.00 kg·ha^−1^, respectively. Statistical analysis indicated no significant difference in yield between the CF and S3 treatments, which suggests that biogas slurry applied during the topdressing stage can partially substitute chemical fertilizer while maintaining stable winter wheat yields.

For the S1 treatment, a tendency toward increased winter wheat yield was observed, yet its yield-promoting effect was less pronounced than that of the S3 treatment. Notably, the S2 treatment led to a reduction in winter wheat yield.

### 3.2. Dynamics of Microbial Relative Abundance

#### 3.2.1. Dynamics of Soil Bacterial Community Composition at Phylum Level

**Figure 3:**
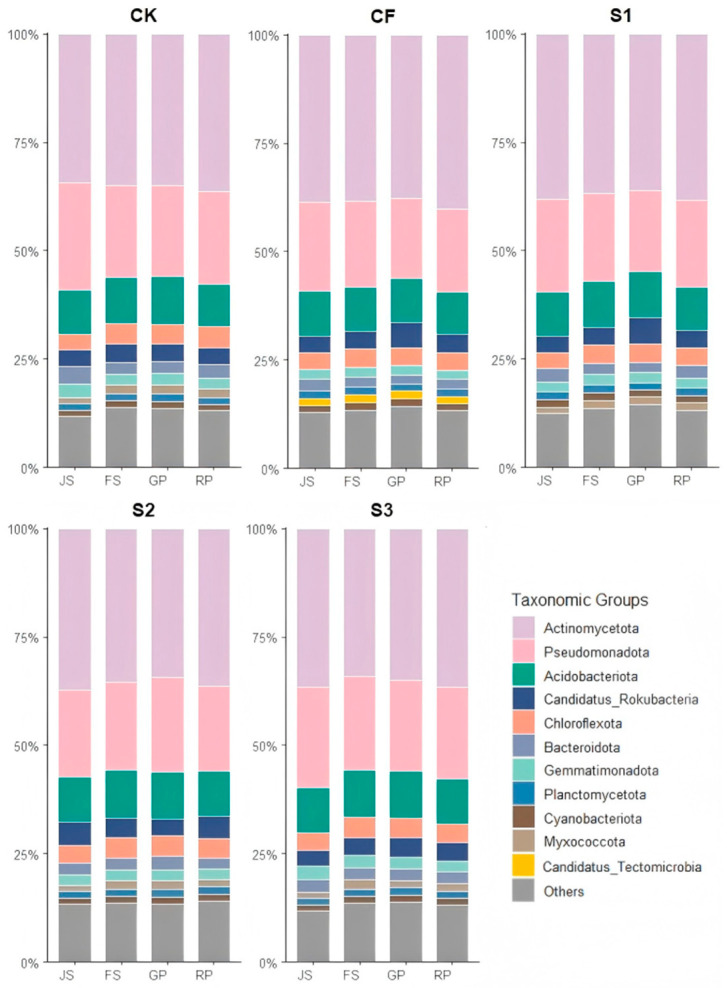
Relative abundance of bacterial communities in different treatments during various periods. JS: jointing stage; FS: flowering stage; GP: grain-filling period; RP: reaping period. CK: Topdressing with water at both JS and GP, CF: Topdressing with chemical fertilizer at both JS and GP, S1: Topdressing with biogas slurry at JS, followed by topdressing with chemical fertilizer at GP, S2: Topdressing with chemical fertilizer at JS, followed by topdressing with biogas slurry at GP, S3: Topdressing with biogas slurry at both JS and GP.

Analysis of bacterial community structures across treatments and growth stages identified Actinomycetota, Pseudomonadota, and Acidobacteriota as the dominant phyla, together accounting for approximately 65% of total bacterial abundance (Figure 3).

Actinomycetota maintained notable stability in the CK treatment (34.30–36.36%), consistently showing the lowest relative abundance among treatments at each sampling stage. Statistically significant differences in Actinomycetota abundance were observed between CK and CF at each growth stage (*p* < 0.05), with CF exhibiting a 4% higher abundance than CK. In the S1 and S3 treatments, Actinomycetota abundance displayed a ‘V-shaped’ pattern, declining initially and then rebounding during the RP, with abundance at the JS and RP stages consistently exceeding intermediate stages by two percentage points. The abundance dynamics of Actinomycetota in S1 closely mirrored those in CF, particularly during the FS and GP. In S2, Actinomycetota abundance showed a unimodal pattern, peaking at 37.16% during JS.

Pseudomonadota abundance in CK initially declined and then stabilized, remaining high across all stages. CF resulted in a consistently lower abundance, decreasing from jointing to ripening. At JS, biogas slurry treatments (S1 and S3) had higher Pseudomonadota abundance than CF, with S3 reaching 23.34%, approaching CK levels, indicating that early biogas slurry application supports the growth of this phylum. S2 showed a significant recovery of Pseudomonadota during GP (21.69%), higher than CF, demonstrating that late-season biogas slurry application stimulates this phylum.

Acidobacteriota abundance in CK remained around 10%, with an initial increase followed by a decline, peaking at 11.08% during GP. The CF treatment exhibited a similar trend but with slightly lower abundance. S1 maintained relatively high Acidobacteriota abundance during FS and GP (10.65% and 10.60%, respectively). S2 and S3 exhibited significantly higher Acidobacteriota abundances during FS compared to CK and CF, with S2 peaking at 10.97% during GP. All biogas slurry treatments showed higher Acidobacteriota abundance (10.05–10.45%) during RP than CK (9.83%) and CF (9.59%), suggesting that biogas slurry helps maintain the Acidobacteriota community size during late growth.

CF treatment notably exhibited significantly higher Candidatus_Tectomicrobia abundance than other treatments, peaking at 1.78% during GP.

In summary, fertilization regimes distinctly affected three key soil bacterial phyla. CK maintained stable abundance; CF increased Actinomycetota but reduced Pseudomonadota and Acidobacteriota, indicating selective pressure from chemical fertilizers. Biogas slurry treatments preserved Pseudomonadota abundance near natural levels with JS application and promoted Acidobacteriota growth with GP application. S3 maintained or increased the abundance of all three phyla by RP, particularly stimulating Acidobacteriota. Compared to chemical fertilizers, biogas slurry outperforms in preserving soil microbial diversity and stability, with JS benefiting Pseudomonadota and GP supporting Acidobacteriota, providing a scientific basis for optimizing slurry application timing.

#### 3.2.2. Dynamics of Soil Fungal Community Composition at Phylum Level

**Figure 4:**
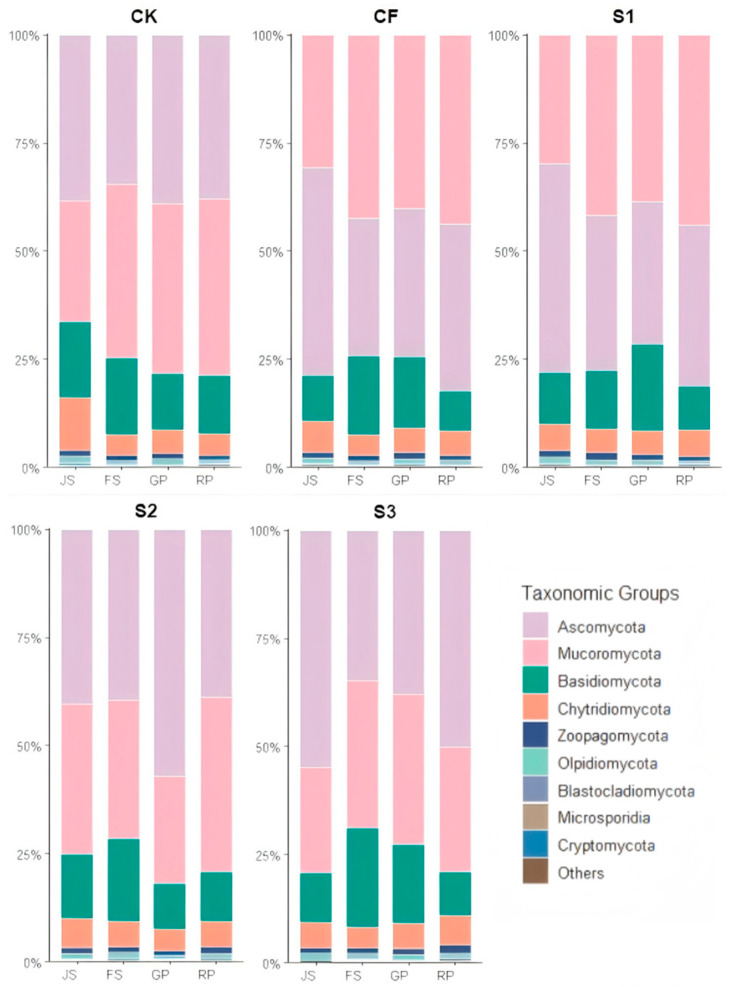
Relative abundance of fungal communities in different treatments during various periods. JS: jointing stage; FS: flowering stage; GP: grain-filling period; RP: reaping period. CK: Topdressing with water at both JS and GP, CF: Topdressing with chemical fertilizer at both JS and GP, S1: Topdressing with biogas slurry at JS, followed by topdressing with chemical fertilizer at GP, S2: Topdressing with chemical fertilizer at JS, followed by topdressing with biogas slurry at GP, S3: Topdressing with biogas slurry at both JS and GP.

Analysis of fungal community structures across treatments and growth stages revealed Ascomycota, Basidiomycota, and Mucoromycota as the dominant phyla, collectively accounting for approximately 90% of total fungal abundance (Figure 4).

The abundance of Ascomycota in CK remained relatively stable across periods, with fluctuations not exceeding 7.07%. In CF, Ascomycota abundance reached a maximum during JS, then sharply declined to 31.77% at FS, followed by a gradual increase. This suggests that chemical fertilizers may strongly stimulate the early Ascomycota community. In S1, Ascomycota abundance was 48.23% during JS, higher than CF, CK, and S2 during the same period. However, its abundance during GP and RP was lower than CK and CF, suggesting that early biogas slurry application can temporarily increase Ascomycota abundance, while subsequent chemical fertilizer application may weaken this sustained effect. In S2, Ascomycota abundance reached 54.14% after additional biogas slurry application during GP, the highest for that period, and remained higher than CK and CF during RP. In S3, Ascomycota abundance during JS and RP was 54.74% and 50.19%, respectively, both the highest in their respective periods.

Basidiomycota abundance in CK remained relatively high (17.45–18.62%) during JS–FS, then decreased to 12.93–13.40% during GP–RP, indicating a correlation between this fungal community and crop growth stages under natural conditions. In CF, abundance peaked at FS (18.35%) but declined markedly to 9.16% at RP, suggesting that chemical fertilizers may disrupt normal Basidiomycota succession. S1 exhibited a peak (20.26%) during GP, a distinct pattern that may reflect a synergistic effect of early biogas slurry and later chemical fertilizer application. In contrast, the S2 trend more closely resembled CK, though at a lower level overall, indicating that late biogas slurry application has limited effects on the Basidiomycota community. S3 reached its highest abundance (23.09%) in FS, significantly higher than in other treatments during this period, suggesting a unique promoting effect of biogas slurry during FS.

The abundance of Mucoromycota in CK showed a slight decline during FS but generally increased over time (Figure 3). CF followed a similar trend but exhibited higher abundance at each period compared to CK, indicating that chemical fertilizers stimulate Mucoromycota proliferation. For biogas slurry top-dressed at JS (S1 and S3), abundances were lower than CK during JS. Similarly, for biogas slurry top-dressed at GP (S2 and S3), abundances were lower than CK during GP. This indicates that biogas slurry can restrict increases in Mucoromycota abundance. Notably, after additional chemical fertilizer application during GP in S1, Mucoromycota abundance exceeded that in CK.

### 3.3. Dynamics of Soil Enzyme Activities

#### 3.3.1. Dynamics of Soil UE Activity

**Figure 5:**
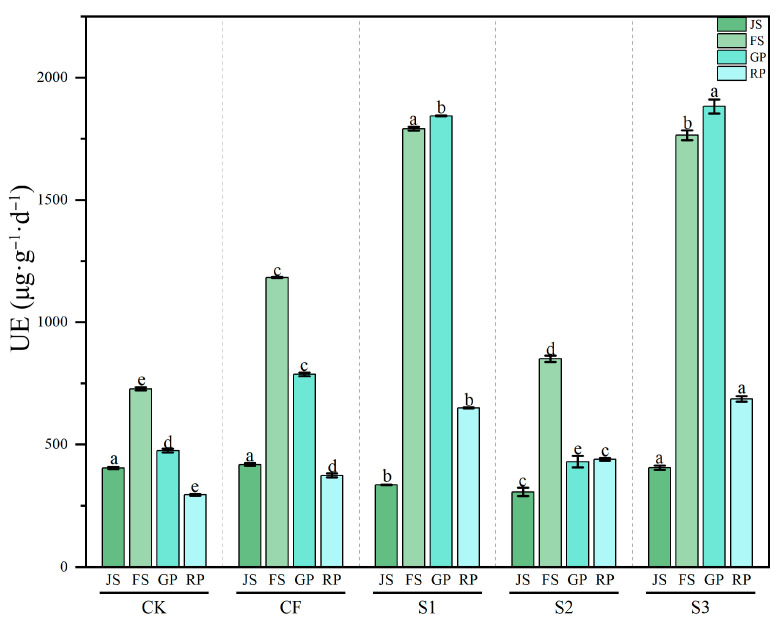
Changes in UE activity across different treatments during various periods. UE: urease; JS: jointing stage; FS: flowering stage; GP: grain-filling period; RP: reaping period. CK: Topdressing with water at both JS and GP, CF: Topdressing with chemical fertilizer at both JS and GP, S1: Topdressing with biogas slurry at JS, followed by topdressing with chemical fertilizer at GP, S2: Topdressing with chemical fertilizer at JS, followed by topdressing with biogas slurry at GP, S3: Topdressing with biogas slurry at both JS and GP. The letters a, b, c, etc. in the picture represent the differences among various treatments within the same period.

UE activity exhibited significant dynamic variation across all treatments (*p* < 0.001) (Figure 5). In the CK treatment, UE activity remained consistently lower than in other treatments, showing an initial increase and then a decline, peaking at the FS (727.61 μg·g^−1^·d^−1^) before decreasing to 294.25 μg·g^−1^·d^−1^ by the RP. The CF treatment displayed higher UE activity overall, reaching 1183.15 μg·g^−1^·d^−1^ at FS, indicating that chemical fertilization markedly stimulated urease activity. Notably, biogas slurry treatments further enhanced UE activity. In S1, UE activity increased sharply at FS, with a further rise to 1843.54 μg·g^−1^·d^−1^ by GP, nearly three times higher than CK. By contrast, the S2 treatment showed a more modest increase, with UE activity at JS and GP even lower than CK. The S3 treatment followed a trend similar to S1, reaching the highest value (1881.90 μg·g^−1^·d^−1^) at GP, exceeding S1. Therefore, compared to CK and CF, S1 and S3 significantly promoted UE activity (*p* < 0.001), whereas S2 did not exhibit such stimulation. Early biogas slurry application was especially critical for enhancing UE activity (*p* < 0.001).

#### 3.3.2. Dynamics of Soil NP Activity

**Figure 6:**
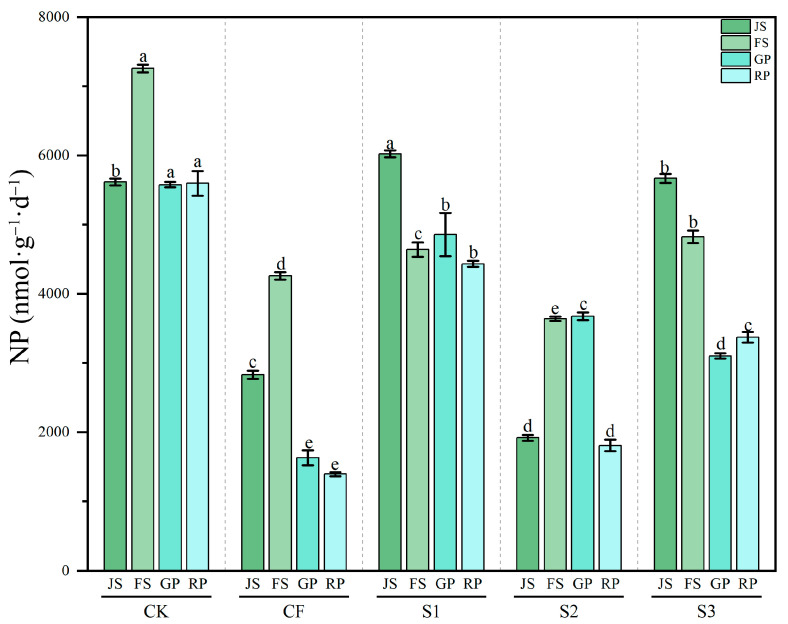
Changes in NP activity across different treatments during various periods. NP: neutral phosphatase; JS: jointing stage; FS: flowering stage; GP: grain-filling period; RP: reaping period. CK: Topdressing with water at both JS and GP, CF: Topdressing with chemical fertilizer at both JS and GP, S1: Topdressing with biogas slurry at JS, followed by topdressing with chemical fertilizer at GP, S2: Topdressing with chemical fertilizer at JS, followed by topdressing with biogas slurry at GP, S3: Topdressing with biogas slurry at both JS and GP. The letters a, b, c, etc. in the picture represent the differences among various treatments within the same period.

NP activity demonstrated significant dynamic variation among treatments (*p* < 0.001) (Figure 6). The CK treatment maintained relatively high NP activity, peaking at 7258.00 nmol·g^−1^·d^−1^ during FS (the highest value observed) and remaining stable at RP (5597.54 nmol·g^−1^·d^−1^), indicating robust phosphorus transformation under natural conditions. NP activity in CF was significantly lower than in CK, decreasing to 1630.20 nmol·g^−1^·d^−1^ at GP and 1394.35 nmol·g^−1^·d^−1^ at RP (a reduction > 70%), likely due to chemical phosphorus fertilizer inhibiting microbial phosphatase secretion. Biogas slurry treatments displayed clear temporal effects on NP activity: S1 and S3 (JS application) showed significantly higher activity at JS (6024.33 and 5669.56 nmol·g^−1^·d^−1^) than CF, approaching CK levels, demonstrating that early slurry application enhances NP activity. S2 maintained similar activity levels between FS-GP and JS-RP, suggesting limited effectiveness of late slurry application in restoring NP activity. S3 showed a decline–recovery pattern, with JS-FS activity significantly higher than at GP-RP. Statistical analysis revealed that both CK and S1 significantly enhanced NP activity (*p* < 0.001). In contrast, CF markedly suppressed NP activity, while S2 did not show a stimulatory effect (*p* < 0.001).

#### 3.3.3. Dynamics of Soil CAT Activity

**Figure 7:**
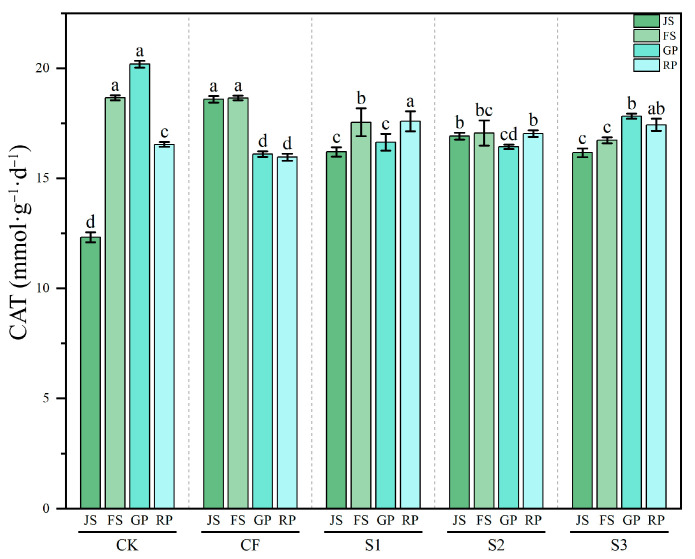
Changes in CAT activity across different treatments during various periods. CAT: catalase; JS: jointing stage; FS: flowering stage; GP: grain-filling period; RP: reaping period. CK: Topdressing with water at both JS and GP, CF: Topdressing with chemical fertilizer at both JS and GP, S1: Topdressing with biogas slurry at JS, followed by topdressing with chemical fertilizer at GP, S2: Topdressing with chemical fertilizer at JS, followed by topdressing with biogas slurry at GP, S3: Topdressing with biogas slurry at both JS and GP. The letters a, b, c, etc. in the picture represent the differences among various treatments within the same period.

CAT activity exhibited distinctive dynamic patterns across treatments (*p* < 0.001) (Figure 7). In CK, CAT activity initially increased and then decreased with crop growth, peaking at GP (20.19 mmol·g^−1^·d^−1^). CF treatment showed similar activity during JS–FS and GP–RP, but with an overall declining trend, suggesting that chemical fertilizer may initially stimulate but later suppress CAT activity. Both S1 and S3 treatments exhibited significantly higher CAT activity than CK at JS, indicating that biogas slurry enhances CAT activity with smaller fluctuations than CK and CF. S2 treatment followed a trend similar to S1, but with even smaller variations, indicating greater stability. Notably, all biogas slurry treatments (S1–S3) maintained significantly higher CAT activity than CF during late growth stages. In particular, S3 showed relatively high activity at GP and RP (17.82 and 17.43 mmol·g^−1^·d^−1^, respectively). These results show that under natural conditions, CAT activity increases with crop growth (*p* < 0.001). Chemical fertilizer application initially stimulates CAT activity but does not sustain it during later periods (*p* < 0.001). In contrast, biogas slurry application provides a more stable protective effect against oxidative stress, especially during advanced crop growth stages (*p* < 0.001).

#### 3.3.4. Dynamics of Soil SC Activity

**Figure 8:**
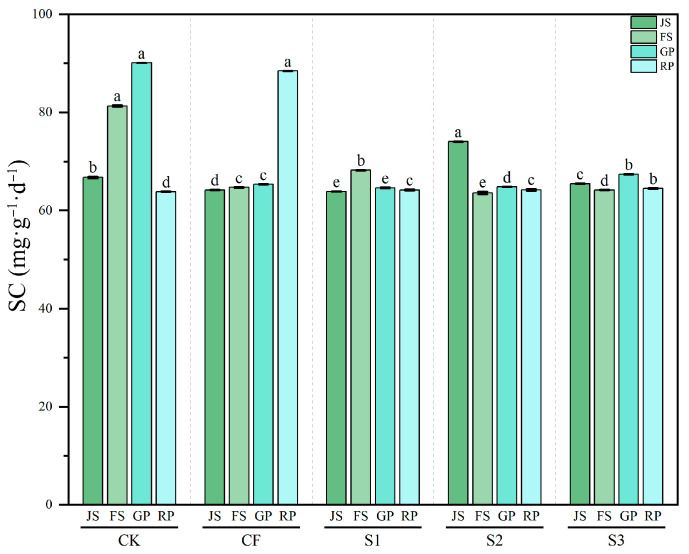
Changes in SC activity across different treatments during various periods. SC: sucrase; JS: jointing stage; FS: flowering stage; GP: grain-filling period; RP: reaping period. CK: Topdressing with water at both JS and GP, CF: Topdressing with chemical fertilizer at both JS and GP, S1: Topdressing with biogas slurry at JS, followed by topdressing with chemical fertilizer at GP, S2: Topdressing with chemical fertilizer at JS, followed by topdressing with biogas slurry at GP, S3: Topdressing with biogas slurry at both JS and GP. The letters a, b, c, etc. in the picture represent the differences among various treatments within the same period.

SC activity exhibited significant dynamic variation across treatments (*p* < 0.001) (Figure 8). Under CK, SC activity initially increased and then decreased, peaking at 90.09 mg·g^−1^·d^−1^ during GP. CF treatment showed a different pattern, with SC activity remaining relatively stable from JS to GP (64.17–65.32 mg·g^−1^·d^−1^), but rising abruptly to 88.46 mg·g^−1^·d^−1^ at RP. The effects of biogas slurry on SC activity were relatively complex. S1 and S3 maintained SC activity within the range of 63.88–68.22 mg·g^−1^·d^−1^ across growth stages, without the pronounced peak observed under CK. S2 showed relatively high SC activity (74.04 mg·g^−1^·d^−1^) at JS, which subsequently declined to levels similar to S1. These results indicated that CF significantly increased SC activity at RP (*p* < 0.001). Moreover, biogas slurry application tended to stabilize SC activity, which may positively affect the stability of soil carbon cycling.

### 3.4. Response of Microbial Communities to Environmental and Enzymatic Drivers

**Figure 9:**
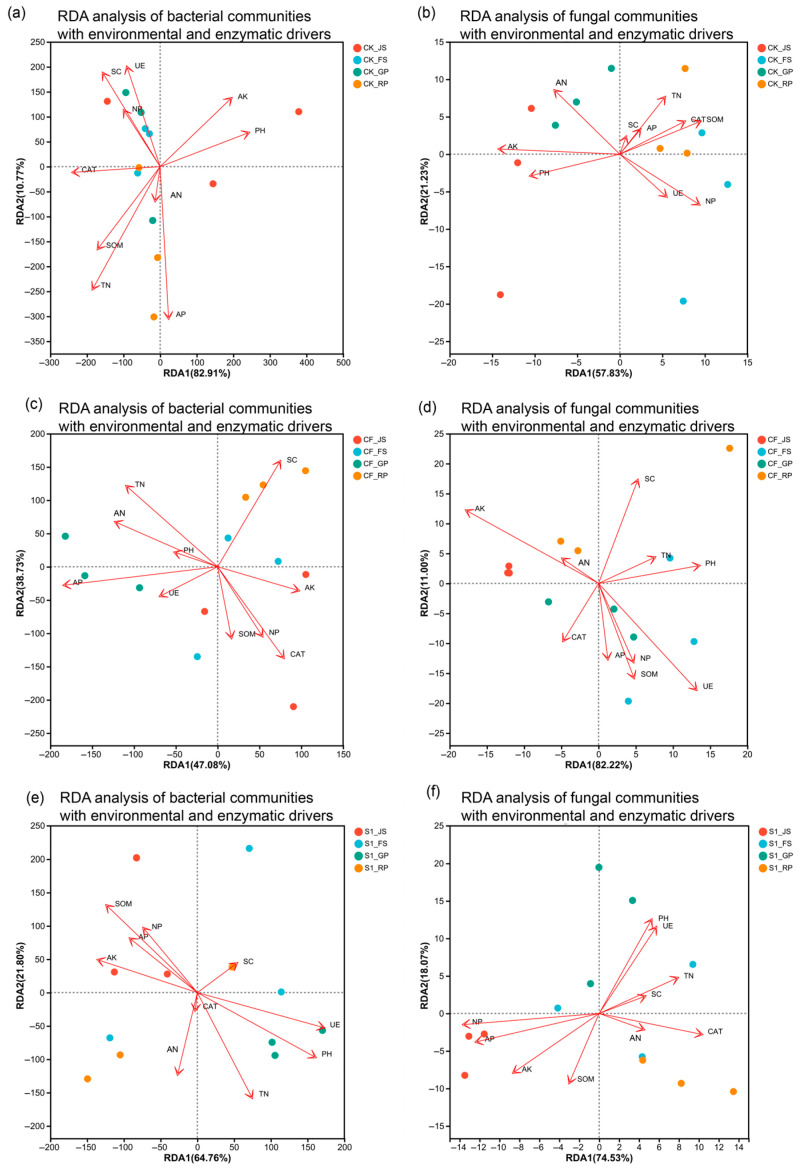

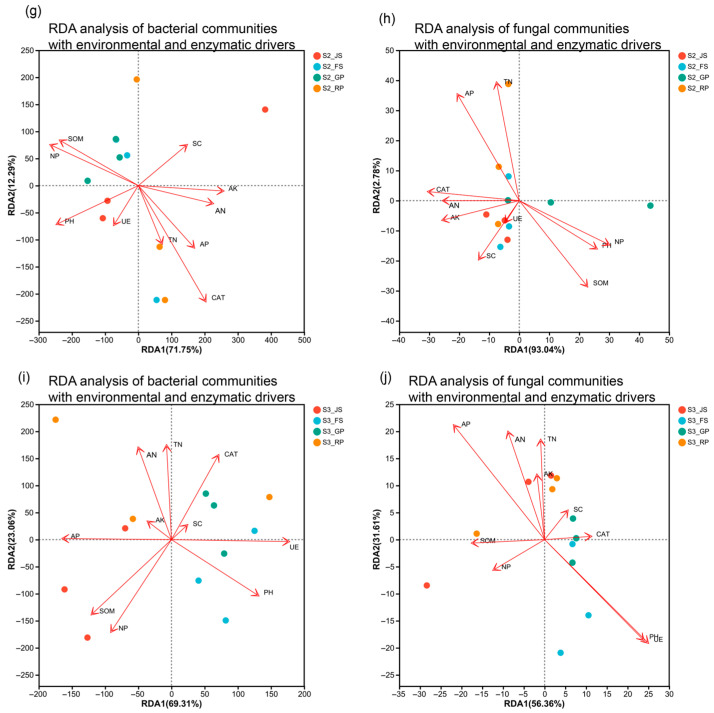
Redundancy Analysis (RDA) of Bacterial and Fungal Communities with Environmental and Enzymatic Drivers. SOM: soil organic matter; TN: total nitrogen; AP: available phosphorus; AK: available potassium; AN: ammonium nitrogen; UE: urease; NP: neutral phosphatase; CAT: catalase; SC: sucrase; JS: jointing stage; FS: flowering stage; GP: grain-filling period; RP: reaping period. CK: Topdressing with water at both JS and GP, CF: Topdressing with chemical fertilizer at both JS and GP, S1: Topdressing with biogas slurry at JS, followed by topdressing with chemical fertilizer at GP, S2: Topdressing with chemical fertilizer at JS, followed by topdressing with biogas slurry at GP, S3: Topdressing with biogas slurry at both JS and GP. Letters (**a**–**j**) label the individual subfigures in sequential order.

Redundancy Analysis (RDA) was used to analyze the variation in soil microbial communities constrained by environmental factors and enzyme activities across different treatments (Figure 9). The first two axes of all RDA models cumulatively explained 79.06% to 95.82% of the variation in microbial communities, indicating that these constrained models effectively captured the primary relationships between environmental factors and community structure.

A systematic comparison of RDA plots for bacterial and fungal communities across five different fertilization treatments revealed that the response patterns of bacterial and fungal communities to environmental factors were fundamentally different, and these differences were precisely regulated by the type and timing of fertilization.

In CK treatment, the bacterial community was primarily positively driven by AK and pH, while the fungal community was dominated by SOM and NP, exhibiting a basic differentiation. The CF treatment further intensified this differentiation: bacteria were instead driven by SC and CAT, while fungi responded strongly to pH and UE.

Importantly, the application of biogas slurry significantly reshaped the driving patterns of microbial communities. Specifically, in S1 treatment, the driving pattern for the bacterial community resembled that of fungi under CF (dominated by UE and pH), suggesting that early organic input can steer the bacterial community toward a state more focused on nitrogen and pH. In contrast, the fungal community in this treatment shifted to being driven by CAT, indicating an enhancement of its organic matter decomposition function. Conversely, S2 treatment presented a different reorganization: its bacterial community was positively driven by AK and CAT, while NP and pH became negative drivers, forming a complex new pattern distinct from both CK and CF. Only SOM, pH, and NP served as positive drivers for fungi in this treatment.

However, S3 treatment ultimately established a stable and highly synergistic new pattern. In this model, the assembly rules of bacterial and fungal communities converged: both were strongly and positively co-driven by UE and pH while simultaneously showing negative correlations with AP and SOM. In the bacterial community, the UE arrow vector was nearly aligned with the RDA1 axis, indicating it as the primary driver shaping bacterial structure. In the fungal community, the UE and pH vectors almost completely overlapped, synergistically forming a powerful composite driving factor. Crucially, in this model, both AN and TN formed acute angles with the RDA2 axis in both bacterial and fungal RDA plots, with the TN arrow almost coinciding with this axis and being the longest.

### 3.5. Correlation Analysis Between Environmental Factors, Microbial Relative Abundance, and Soil Enzyme Activities

**Figure 10:**
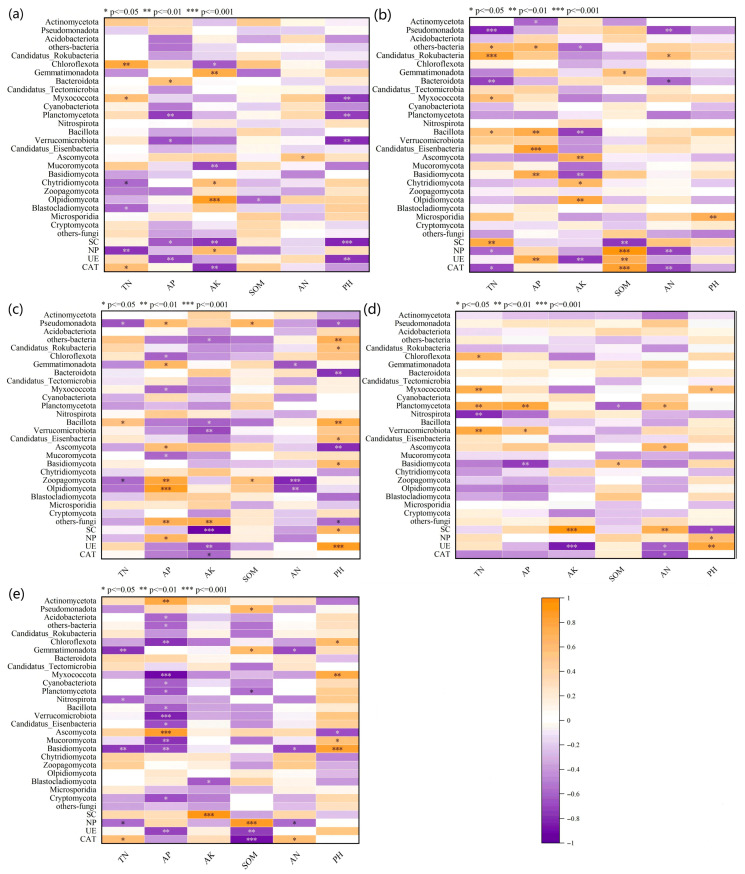
Spearman correlation analysis of environmental factors, enzyme activities, and microbial communities under different treatments. (**a**–**e**) represent CK, CF, S1, S2, and S3, respectively. SOM: soil organic matter; TN: total nitrogen; AP: available phosphorus; AK: available potassium; AN: ammonium nitrogen; UE: urease; NP: neutral phosphatase; CAT: catalase; SC: sucrase.

The differential regulation of soil microorganisms and enzyme activities by biogas slurry application suggests that its effects result not only from the direct input of organic nutrients but also from indirect reshaping of the microbe–enzyme interaction network by altering soil environmental factors such as pH and TN (Figure 10a–e). To clarify these processes, Spearman correlation analyses were performed between key environmental factors, microbial community structure, and enzyme activities. Compared with the CK and CF treatments, S1 and S2 altered the significant correlations between TN and NP, as well as between TN and CAT. These treatments also modified significant correlations between TN and most microbial taxa. In S3 treatment, AP exhibited significant negative correlations with several microorganisms (*p* ≤ 0.01) but showed a highly significant positive correlation with Ascomycota (*p* ≤ 0.001). Notably, biogas slurry treatments (S1, S2, S3) substantially modified the significant relationships between AK and microorganisms. Specifically, only in S1 did AK display a significant relationship with a single microbial taxon, while no significant relationships were found in S2 and S3. In both CF and S3 treatments, SOM showed a highly significant positive correlation with NP (*p* ≤ 0.001), indirectly suggesting that both biogas slurry and chemical fertilizer can enhance the effect of organic matter on AP. However, SOM displayed different highly significant relationships with CAT in these two treatments. In CF, AN exhibited significant negative correlations with both NP and CAT activity (*p* ≤ 0.01), whereas in S2, AN showed a significant positive correlation with SC activity (*p* ≤ 0.01). Relationships with pH varied across treatments. In S3, pH did not show a significant correlation with any microorganism. Therefore, the application of biogas slurry significantly influences the interaction network between microbial communities and enzyme activities by dynamically regulating key soil environmental factors such as TN, AP, AK, and SOM. Its effects rely not only on direct nutrient input but also on the indirect induction of synergistic or antagonistic microbial–enzyme responses by altering soil physicochemical properties. These results reveal the multi-pathway mechanism of biogas slurry in regulating soil micro-ecology.

### 3.6. Relationships Between Microbial Relative Abundance and Enzyme Activities

**Figure 11:**
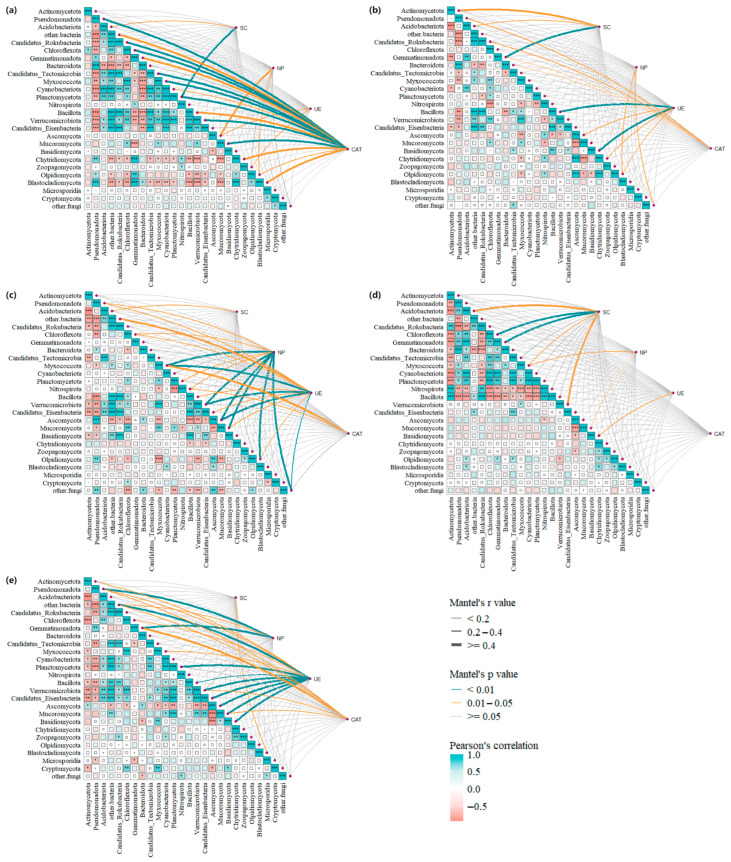
Pearson correlation and partial Mantel test analysis showed the relationship between microbial communities and enzymes. (**a**–**e**) represent CK, CF, S1, S2, and S3, respectively. UE: urease; NP: neutral phosphatase; CAT: catalase; SC: sucrase; * represents significant at *p* < 0.05; ** represents significant at *p* < 0.01; *** represents significant at *p* < 0.001.

The differential effects of fertilization treatments on soil microbial community structure and enzyme activities (Figure 3, Figure 4, Figure 5, Figure 6, Figure 7 and Figure 8) are likely mediated through alterations in soil physicochemical properties, which subsequently influence microbe–enzyme interaction processes (Figure 10a–e). To assess these relationships, association characteristics between key microbial taxa and enzyme activities were quantified under each treatment using Pearson correlation analysis and Mantel tests (Figure 11a–e).

In CK treatment (a), Planctomycetota exhibited a highly significant, strong positive correlation with SC activity (r = 0.502, *p* = 0.003). Acidobacteriota also showed a significant moderately strong positive correlation with SC activity (r = 0.211, *p* = 0.04), whereas no significant correlation was observed between SC activity and fungi. NP and UE activities did not show significant correlations with bacteria. NP activity displayed significant moderately strong correlations with the fungal phyla Ascomycota (r = 0.415) and Olpidiomycota (r = 0.346, *p* < 0.05). Similarly, UE activity was significantly and moderately correlated with Ascomycota (r = 0.246) and Zoopagomycota (r = 0.289, *p* < 0.05). In contrast, CAT activity demonstrated positive correlations with most bacterial groups, showing highly significant strong positive correlations with Pseudomonadota, Candidatus_Rokubacteria, Chloroflexota, Candidatus_Tectomicrobia, Planctomycetota, Myxococcota, and Verrucomicrobiota (r ≥ 0.4, *p* < 0.01). The fungal phylum Mucoromycota (r = 0.646) also showed a highly significant strong positive correlation with CAT activity (*p* < 0.01).

In CF treatment (b), SC activity did not show a significant relationship with fungi, but Gemmatimonadota was identified as an important driver for SC activity (*p* = 0.001). NP activity did not show a significant correlation with fungi, whereas UE activity exhibited a highly significant strong positive correlation with Bacillota (r ≥ 0.4, *p* < 0.01). CAT activity did not display a significant relationship with fungi, nor did it generally show highly significant correlations with bacteria. However, CAT was significantly correlated with Pseudomonadota, Candidatus_Rokubacteria, Gemmatimonadota, and Bacteroidota (*p* < 0.05).

In S1 treatment (c), SC activity remained uncorrelated with fungi, but correlations between NP and UE activities and certain bacterial taxa reached highly significant levels (*p* < 0.01). Specifically, Myxococcota and Verrucomicrobiota exhibited highly significant strong positive correlations with both NP and UE activities (r ≥ 0.4, *p* < 0.01). As in the CF treatment, CAT activity did not show a significant relationship with fungi or a highly significant relationship with bacteria overall.

In S2 treatment (d), SC activity continued to show no significant correlation with fungi but was highly positively correlated with Chloroflexota (r = 0.412) and Gemmatimonadota (r = 0.543). NP activity was significantly positively correlated with Myxococcota and the fungal phylum Mucoromycota (*p* < 0.05). UE did not show significant correlations with any bacteria, and CAT did not exhibit significant relationships with any bacteria or fungi.

In S3 treatment (e), only the fungal phylum Blastocladiomycota (r = 0.371) showed a significant moderately strong positive correlation with SC activity (*p* = 0.015). Pseudomonadota (r = 0.425) and Gemmatimonadota (r = 0.617) were identified as the main drivers of NP activity under S3 (*p* < 0.01). NP activity did not show significant correlations with fungi. UE activity demonstrated highly significant strong positive correlations with Planctomycetota, Verrucomicrobiota, and Candidatus_Eisenbacteria (r ≥ 0.4, *p* < 0.01), as well as with Ascomycota, Mucoromycota, and Basidiomycota (r ≥ 0.4, *p* < 0.01). Myxococcota (r = 0.248) and Chloroflexota (r = 0.200) also exhibited significant moderately positive correlations with UE activity. CAT activity was significantly moderately correlated with Pseudomonadota, Verrucomicrobiota, and Mucoromycota (0.2 ≤ r < 0.4, *p* < 0.05) and strongly correlated with Gemmatimonadota (r ≥ 0.4).

## 4. Discussion

### 4.1. Response of Soil Microorganisms to Biogas Slurry Application

Soil pH can impose direct physiological stress on bacterial cells, and within specific pH ranges, different bacterial taxa display marked differences in growth adaptability [38,39,40]. Soils with higher pH generally show greater relative abundance of Actinomycetota and lower abundance of Acidobacteriota [41,42]. In this study, the relative abundance of Acidobacteriota was higher in the biogas slurry treatments compared to CK and CF, suggesting that biogas slurry may create a locally acidic environment via organic matter decomposition. Factors beyond pH, such as soil moisture and SOM, also influence bacterial community patterns. Goldfarb et al. [43] reported that the relative abundance of several taxa, primarily within Pseudomonadota and Actinomycetota, increased with labile organic substrates such as glycine and sucrose. The greater relative abundance of Pseudomonadota observed in S1 and S3 compared to CF, as well as the resurgence of this group in S2 treatment at the GP, indicate that biogas slurry supplies the organic compounds needed by Pseudomonadota.

Ascomycota exhibited the most complex response pattern. The transient peak in Ascomycota abundance during the JS in the CF treatment reflects a positive correlation between chemical nitrogen fertilizer and Ascomycota, but this effect was not sustained, as evidenced by the sharp decline at FS. This observation is consistent with the study by Ramirez et al. [44], who reported that inorganic nitrogen inhibits microbial activity, and that high nitrogen levels can suppress Ascomycota abundance [45]. By contrast, biogas slurry treatments (especially S2 and S3) demonstrated a more stable promoting effect, likely because the complex organic compounds in biogas slurry are better suited to the degradation characteristics of Ascomycota [46]. Basidiomycota play a significant role in the carbon cycle [47]. Their abundance is strongly influenced by soil organic carbon content, as increased organic carbon provides ample substrate for their growth and reproduction. However, Feng [48] found that the application of chemical fertilizers alone accelerates the depletion of organic carbon, reduces the available substrate, and consequently leads to a decline in the abundance of Basidiomycota. This aligns with the present finding that Basidiomycota relative abundance was higher in S3 than CF. Mora-Salguero et al. [49] observed minimal changes in Mucoromycota abundance following pig slurry application, indicating a limited capacity of biogas slurry to modulate its abundance.

In summary, the modulation of the soil microbial community by biogas slurry is primarily driven by four interconnected factors: (1) the introduction of complex organic matter that serves as a preferred substrate for copiotrophic bacteria (e.g., Pseudomonadota) and saprophytic fungi; (2) the acidification of soil during decomposition, which selects for acid-tolerant taxa like Acidobacteriota; (3) provision of a slow-release nitrogen source that sustains microbial activity without the inhibitory effects associated with high concentrations of inorganic fertilizer; and (4) the overarching enrichment of SOC, which establishes a more abundant and diverse energy base for the entire microbial network. This multi-faceted manipulation drives a shift towards a more robust and self-sustaining soil ecosystem.

The application of biogas slurry restructured the soil microbial community. This shift was marked by increased stability of key fungal phyla like Ascomycota, alongside a rise in functionally important bacterial groups, particularly within Pseudomonadota. This transformation of the microbial community facilitates a more resilient and self-regulating soil ecosystem. This shift represents a fundamental transition from a community dependent on exogenous chemical nitrogen inputs to one sustained by complex organic substrates. Such microbial network robustness constitutes a primary indicator of soil health and ecological functionality. In the context of sustainable agricultural systems, the strategic management of soils to promote these intricate microbial consortia offers substantial benefits, including reduced dependency on synthetic fertilizers, optimization of biogeochemical nutrient cycling processes, and enhancement of long-term agricultural productivity through improved soil biological fertility.

### 4.2. Response of Soil Enzymes to Biogas Slurry Application

SOM and organic residues decompose into a range of products under the action of microorganisms and enzymes, providing the energy and material foundation for microbial and enzymatic activity. This process directly or indirectly affects microbial growth and soil enzyme activity [50]. In addition, organic matter influences microbial proliferation and enzyme secretion by altering the physical properties of soil, such as porosity, aggregate structure, and water-holding capacity [51]. UE activity is crucial for the nitrogen cycle by catalyzing urea hydrolysis, regulating nitrogen supply after fertilization and influencing plant nitrogen uptake [52,53]. SC is closely associated with soil carbon cycling, primarily participating in the decomposition of active organic carbon fractions [54].

A previous study has shown that increased SOM and available nutrient content enhance UE and SC activity, and that enhanced enzyme activity in turn accelerates SOM decomposition and nutrient transformation [55]. This experiment corroborated these conclusions, with S1 and S3 treatments displaying stronger stimulation of UE activity and maintaining elevated levels, especially during GP. Biogas slurry treatments also supported carbon cycle stability. These results indicate that biogas slurry application more effectively promotes long-term soil microbial activity and enzyme stability than sole chemical fertilizer use. Similarly, Kandeler and Eder [56] found that urease activity increased with the application of cow slurry.

Biogas slurry contains abundant readily available nutrients, easily decomposable nitrogenous compounds, and a large number of microorganisms that promote enzyme secretion [57,58]. Zhang [59] has found that biogas slurry enhances soil enzyme activities such as NP more effectively than chemical fertilizers. In addition, biogas slurry is rich in humus and SOM [60,61], both of which stimulate CAT activity. The S1 and S3 treatments in this study were consistent with previous findings, showing increased NP activity compared to CF. Moreover, CAT activity during JS was significantly higher in S1 and S3 than CK.

The persistent enhancement of critical soil enzyme activities following biogas slurry application directly correlates with improved ecosystem functioning in agricultural soils. Through continuous support of the microbial catalysts driving carbon (SC), nitrogen (UE), and phosphorus (NP) biogeochemical cycles, biogas slurry facilitates the closure of nutrient loops and sustains soil fertility dynamics. This phenomenon represents a core principle of sustainable agricultural intensification. From an environmental sustainability perspective, organic amendments such as biogas slurry can mitigate these issues by promoting intrinsic biochemical processes. This approach fosters the development of climate-resilient and sustainable agricultural production systems.

### 4.3. Synergistic Relationship Between Microorganisms and Enzymes Under Biogas Slurry Application

This study systematically demonstrated the association patterns between soil microbial communities and enzyme activities under different fertilization treatments, indicating that fertilization mediates microbe–enzyme interactions primarily by altering soil physicochemical properties. Notably, in the CK treatment, Planctomycetota and Acidobacteriota strongly influenced SC activity, while CAT activity exhibited highly significant positive correlations with several bacterial phyla (such as Pseudomonadota, Chloroflexota) and the fungal phylum Mucoromycota. However, the microbe–enzyme interaction network is highly complex. For instance, in CF, Gemmatimonadota was a key driver for SC activity, whereas CAT activity did not show significant associations with fungi. This difference suggests that fertilization strategies can influence enzyme function by selectively modulating specific microbial taxa and thus nutrient cycling.

Previous studies have supported a close link between microbial community structure and enzyme activities. Han et al. [62] found that Planctomycetota displayed a highly significant positive correlation with SC activity after foliar spraying of potassium sorbitol chelate, consistent with our CK findings. He [63] found that green manure incorporation significantly enhanced CAT activity, and CAT was positively correlated with Gemmatimonadota abundance, suggesting a role for Gemmatimonadota in promoting CAT secretion and protecting plants from hydrogen peroxide damage. The present findings for S3 are consistent with this observation. Zhang [64] observed a significant positive correlation between Gemmatimonadota and SC in non-rhizosphere soil, aligning with the CF and S2 treatments here. The current study further demonstrated that replacing chemical fertilizers with biogas slurry (S1–S3) markedly alters microbial–enzyme interaction patterns. For instance, S3 showed highly significant positive correlations between UE activity and Planctomycetota, Verrucomicrobiota, and several fungal phyla, suggesting that complete substitution with biogas slurry can enhance UE functionality through strengthened bacterial–fungal co-occurrence networks. Importantly, previous studies have shown that organic manure can enhance microbial interactions, thereby contributing to improved soil health [65,66]. Biogas slurry also exerts a positive effect on soil health when used as a fertilizer, though its application concentration should not be excessively high. In their research on biogas slurry as a fertilizer, Meng et al. [67] observed that biogas slurry with a concentration of ≥10% could inhibit seed germination and root elongation, with the germination rate decreasing from 87.6% to 2.4%. However, biogas slurry at concentrations of 50% and 100% promoted crop growth due to its sufficient nutrient supply. Furthermore, in the long-term application of biogas slurry with high concentrations—particularly at 100% concentration—potential salt accumulation and harmful effects on rhizosphere bacteria in the soil were detected. Mdlambuzi et al. [68] investigated the application of proportionally combined biogas slurry and chemical fertilizers to maize. Their results indicated that soil pH value and total nitrogen content increased with the rising proportion of biogas slurry, while the contents of soil organic carbon, extractable phosphorus, and exchangeable potassium were relatively higher in the biogas slurry-treated groups.

Although this study employed a multivariate approach to quantify the relationships between microorganisms and enzyme activities under fertilization conditions, several limitations remain. First, the inherent differences in biogas slurry feedstock lead to high variability in its composition. Second, this study did not evaluate the potential ecological risks of long-term slurry application. These unassessed risks include the dissemination of antibiotic resistance genes and pathogenic microorganisms, as well as the accumulation of heavy metals and trace elements. Furthermore, as the experiment was conducted over one year with only winter wheat, it could not reflect the long-term effects of the fertilization practices. Additionally, the lack of absolute microbial abundance data limits our ability to discern true shifts in microbial population sizes. Future research should aim to systematically explore the mechanisms and ecological risks in microbe–enzyme interactions driven by biogas slurry. To this end, long-term field experiments are needed, utilizing slurries from diverse sources. These studies should employ a suite of multidimensional techniques, such as metagenomics, contaminantomics, and absolute microbial quantification.

## 5. Conclusions

This study systematically investigated the differential effects of top-dressed biogas slurry application at distinct winter wheat growth stages (JS, GP) on soil enzyme–microbe interactions. The findings demonstrate that biogas slurry application significantly altered (1) the activities of key soil enzymes—UE, NP, SC, and CAT; (2) the community structure of major microbial functional groups such as Actinomycetota, Pseudomonadota, and Ascomycota; and (3) ultimately reshaped microbe–enzyme interaction networks by modulating soil environmental factors (e.g., TN, AP, and pH). Specifically, biogas slurry application at JS (S1 and S3 treatments) significantly enhanced both Pseudomonadota abundance and UE activity, thereby stimulating soil nitrogen transformation. In contrast, application during GP (S2 and S3 treatments) markedly increased Acidobacteriota abundance and NP activity, thus promoting phosphorus cycling. Furthermore, biogas slurry application significantly increased CAT activity, especially during the later stages of crop growth, indicating its potential to provide enhanced protection against oxidative stress. Distinct correlation patterns between microbial taxa and enzyme activities were observed under different fertilization treatments. Substituting biogas slurry for chemical fertilizer substantially modified the microbial–enzyme interaction network. In CK, enzyme activities were primarily driven by bacteria, as indicated by the highly significant correlation between Planctomycetota and SC activity (*p* < 0.01). In CF, Gemmatimonadota emerged as the primary driver of SC activity, but overall microbial association strength was weakened. Biogas slurry application at JS (S1) significantly enhanced the regulatory roles of Myxococcota and Verrucomicrobiota on both NP and UE activities. By contrast, GP application (S2) primarily strengthened the influence of Chloroflexota and Gemmatimonadota on SC activity. Notably, the full-cycle biogas slurry treatment (S3) exhibited the most complex microbe–enzyme network, with both bacterial taxa (Pseudomonadota and Gemmatimonadota) showing highly significant correlations with NP activity and fungal communities (e.g., Ascomycota and Mucoromycota) displaying strong positive associations with UE activity. Overall, these results indicate that biogas slurry application optimizes microbe–enzyme interactions through dynamic regulation of soil physicochemical properties, thereby enhancing soil nutrient cycling and carbon–nitrogen metabolism.

Yield analysis revealed that CF treatment achieved the highest yield (9632.00 kg·ha^−1^), with no significant difference from S3 treatment (9461.33 kg·ha^−1^). This indicates that biogas slurry effectively substitutes chemical fertilizers during topdressing to maintain stable winter wheat yields. Consequently, these results provide robust evidence for adopting biogas slurry as a chemical fertilizer alternative.

To maximize fertilizer efficiency and ecological benefits, field management strategies should prioritize dual biogas slurry applications at jointing (JS) and grain-filling (GP) stages where feasible. This approach enhances soil nitrogen–phosphorus cycling and promotes stable, synergistic microbial communities. When single-stage application is necessary, JS stage application is critical to ensure adequate nitrogen supply during stem elongation and spike development.

## Figures and Tables

**Table 1:** Soil enzymes and their corresponding enzyme functional classification, abbreviation, and enzyme function.

Name of Soil Enzyme	Functional Classification	Abbreviation	Enzyme Function
Sucrase	Hydrolysis	SC	It can catalyze the hydrolysis of sucrose into glucose and fructose [34]
Catalase	Oxidation	CAT	It can degrade the buildup of H_2_O_2_ caused by soil organisms’ metabolic activity [35]
Urease	Hydrolysis	UE	It can catalyzes the hydrolysis of urea [36]
Neutral phosphatase	Hydrolysis	NP	It can release P contained in organic matter for reuse by living organisms [37]

## Data Availability

The raw data supporting the conclusions of this article will be made available by the authors on request.

## References

[1] Ndabankulu K., Egbewale S.O., Tsvuura Z., Magadlela A. (2022). Soil Microbes and Associated Extracellular Enzymes Largely Impact Nutrient Bioavailability in Acidic and Nutrient Poor Grassland Ecosystem Soils. Sci. Rep..

[2] Ayyar S., Appavoo S., Basker M., Pandiyarajan P., Kavimani R. (2019). Effect of Zinc and Microbial Inoculation on Soil Enzyme Activities for Maize (*Zea mays* L.) in Black Soil. Int. J. Curr. Microbiol. Appl. Sci..

[3] Kumari A., Dash M., Singh S.K., Jagadesh M., Mathpal B., Mishra P.K., Pandey S.K., Verma K.K. (2023). Soil Microbes: A Natural Solution for Mitigating the Impact of Climate Change. Environ. Monit. Assess..

[4] Banerjee S., Walder F., Büchi L., Meyer M., Held A.Y., Gattinger A., Keller T., Charles R., van der Heijden M.G. (2019). Agricultural Intensification Reduces Microbial Network Complexity and the Abundance of Keystone Taxa in Roots. ISME J..

[5] Philippot L., Spor A., Hénault C., Bru D., Bizouard F., Jones C.M., Sarr A., Maron P.-A. (2013). Loss in Microbial Diversity Affects Nitrogen Cycling in Soil. ISME J..

[6] Delgado-Baquerizo M., Eldridge D.J., Ochoa V., Gozalo B., Singh B.K., Maestre F.T. (2017). Soil Microbial Communities Drive the Resistance of Ecosystem Multifunctionality to Global Change in Drylands across the Globe. Ecol. Lett..

[7] Chai Y., Li X., Li C., Ma Y., Song Z., Gao P., Ba Y., Wei W. (2024). Soil Moisture Regulates Soil-Microbe-Enzyme Stoichiometries during Recovery Succession in Patchily Degraded Alpine Meadows on the Qinghai-Tibet Plateau. Ecol. Eng..

[8] Lacava P.T., Machado P.C., De Andrade P.H.M., Maheshwari D.K., Dheeman S. (2021). Phosphate Solubilization by Endophytes from the Tropical Plants. Endophytes: Mineral Nutrient Management, Volume 3.

[9] Rao D.L.N., Aparna K., Mohanty S.R. (2019). Microbiology and Biochemistry of Soil Organic Matter, Carbon Sequestration and Soil Health. Indian J. Fertil..

[10] Costa O.Y.A., Raaijmakers J.M., Kuramae E.E. (2018). Microbial Extracellular Polymeric Substances: Ecological Function and Impact on Soil Aggregation. Front. Microbiol..

[11] Li Y., Wang C., Wu J., Zhang Y., Li Q., Liu S., Gao Y. (2023). The Effects of Localized Plant–Soil–Microbe Interactions on Soil Nitrogen Cycle in Maize Rhizosphere Soil under Long-Term Fertilizers. Agronomy.

[12] El-Akhdar I., Shabana M.M.A., El-Khateeb N.M.M., Elhawat N., Alshaal T. (2024). Sustainable Wheat Cultivation in Sandy Soils: Impact of Organic and Biofertilizer Use on Soil Health and Crop Yield. Plants.

[13] Mgxaji Y., Mutengwa C.S., Mukumba P., Dzvene A.R. (2025). Biogas Slurry as a Sustainable Organic Fertilizer for Sorghum Production in Sandy Soils: A Review of Feedstock Sources, Application Methods, and Agronomic Impacts. Agronomy.

[14] De França A.A., Von Tucher S., Schmidhalter U. (2021). Effects of Combined Application of Acidified Biogas Slurry and Chemical Fertilizer on Crop Production and N Soil Fertility. Eur. J. Agron..

[15] Kumar S., Malav L.C., Malav M.K., Khan S.A. (2015). Biogas Slurry: Source of Nutrients for Eco-Friendly Agriculture. Int. J. Extensive Res..

[16] Niyungeko C., Liang X., Liu C., Zhou J., Chen L., Lu Y., Tiimub B.M., Li F. (2020). Effect of Biogas Slurry Application on Soil Nutrients, Phosphomonoesterase Activities, and Phosphorus Species Distribution. J. Soils Sediments.

[17] Moeskops B., Sukristiyonubowo, Buchan D., Sleutel S., Herawaty L., Husen E., Saraswati R., Setyorini D., De Neve S. (2010). Soil Microbial Communities and Activities under Intensive Organic and Conventional Vegetable Farming in West Java, Indonesia. Appl. Soil Ecol..

[18] Basha S.J., Basavarajappa R., Shimalli G., Babalad H.B. (2017). Soil Microbial Dynamics and Enzyme Activities as Influenced by Organic and Inorganic Nutrient Management in Vertisols under Aerobic Rice Cultivation. J. Environ. Biol..

[19] Plaza C., Hernández D., García-Gil J.C., Polo A. (2004). Microbial Activity in Pig Slurry-Amended Soils under Semiarid Conditions. Soil Biol. Biochem..

[20] Feng W., Guan T., Wang X., Zhu Y., Guo T. (2011). Effects of Combined Application of Biogas Slurry and Chemical Fertilizer on Winter Wheat Rhizosphere Soil Microorganisms and Enzyme Activities. Chin. J. Appl. Ecol..

[21] Yadav R., Sudhishri S., Khanna M., Lal K., Dass A., Kushwaha H.L., Bandyopadhyay K., Suman A., Singh A., Singh R.K. (2024). A Greener Approach to Spinach Farming: Drip Nutrigation with Biogas Slurry Digestate. Agronomy.

[22] Abbas A., Naveed M., Azeem M., Yaseen M., Ullah R., Alamri S., Ain Farooq Q.U., Siddiqui M.H. (2020). Efficiency of Wheat Straw Biochar in Combination with Compost and Biogas Slurry for Enhancing Nutritional Status and Productivity of Soil and Plant. Plants.

[23] Murase J., Hida A., Ogawa K., Nonoyama T., Yoshikawa N., Imai K. (2015). Impact of Long-Term Fertilizer Treatment on the Microeukaryotic Community Structure of a Rice Field Soil. Soil Biol. Biochem..

[24] Sapp M., Harrison M., Hany U., Charlton A., Thwaites R. (2015). Comparing the Effect of Digestate and Chemical Fertiliser on Soil Bacteria. Appl. Soil Ecol..

[25] Sabir M.S., Shahzadi F., Ali F., Shakeela Q., Niaz Z., Ahmed S. (2021). Comparative Effect of Fertilization Practices on Soil Microbial Diversity and Activity: An Overview. Curr. Microbiol..

[26] Abubaker J., Cederlund H., Arthurson V., Pell M. (2013). Bacterial Community Structure and Microbial Activity in Different Soils Amended with Biogas Residues and Cattle Slurry. Appl. Soil Ecol..

[27] Yu X.-Y., Zhu Y.-J., Jin L., Wang B.-T., Xu X., Zou X., Ruan H.-H., Jin F.-J. (2022). Contrasting Responses of Fungal and Bacterial Communities to Biogas Slurry Addition in Rhizospheric Soil of Poplar Plantations. Appl. Soil Ecol..

[28] Atav V., Yüksel O., Namlı A., Gürbüz M.A. (2024). Biogas Liquid Digestate Application: Influence on Soil Microbial Biomass and CO_2_ Respiration. J. Mater. Cycles Waste Manag..

[29] Gryń G., Gryń G., Paluszak Z., Olszewska H., Keutgen A.J. (2020). Chemical and Microbiological Properties of Luvisol after Addition of Post-Fermentation Residue. J. Elem..

[30] Johansson L.H., Håkan Borg L.A. (1988). A Spectrophotometric Method for Determination of Catalase Activity in Small Tissue Samples. Anal. Biochem..

[31] Guo H., Yao J., Cai M., Qian Y., Guo Y., Richnow H.H., Blake R.E., Doni S., Ceccanti B. (2012). Effects of Petroleum Contamination on Soil Microbial Numbers, Metabolic Activity and Urease Activity. Chemosphere.

[32] Zhang F., Qiao Z., Yao C., Sun S., Liu W., Wang J. (2021). Effects of the Novel HPPD-Inhibitor Herbicide QYM201 on Enzyme Activity and Microorganisms, and Its Degradation in Soil. Ecotoxicology.

[33] Liu B., Wang S., Wang J., Zhang X., Shen Z., Shi L., Chen Y. (2020). The Great Potential for Phytoremediation of Abandoned Tailings Pond Using Ectomycorrhizal Pinus Sylvestris. Sci. Total Environ..

[34] Farooq T.H., Kumar U., Mo J., Shakoor A., Wang J., Rashid M.H.U., Tufail M.A., Chen X., Yan W. (2021). Intercropping of Peanut–Tea Enhances Soil Enzymatic Activity and Soil Nutrient Status at Different Soil Profiles in Subtropical Southern China. Plants.

[35] Türkay F.Ş.H., Durmuş M., Yakupoğlu T. (2024). Exploring Catalase Activity as A Biological Indicator in Degraded Soils. Anadolu Tarım Bilim. Derg..

[36] Dharmakeerthi R.S., Thenabadu M.W. (1996). Urease activity in soils: A review. J. Natl. Sci. Found. Sri Lanka.

[37] Rocabruna P.C., Domene X., Preece C., Peñuelas J. (2024). Relationship among Soil Biophysicochemical Properties, Agricultural Practices and Climate Factors Influencing Soil Phosphatase Activity in Agricultural Land. Agriculture.

[38] Kowalchuk G.A., Stephen J.R., Boer W.D., Prosser J.I., Embley T.M., Woldendorp J.W. (1997). Analysis of Ammonia-Oxidizing Bacteria of the Beta Subdivision of the Class Proteobacteria in Coastal Sand Dunes by Denaturing Gradient Gel Electrophoresis and Sequencing of PCR-Amplified 16S Ribosomal DNA Fragments. Appl. Environ. Microbiol..

[39] Lauber C.L., Strickland M.S., Bradford M.A., Fierer N. (2008). The Influence of Soil Properties on the Structure of Bacterial and Fungal Communities across Land-Use Types. Soil Biol. Biochem..

[40] McCaig A.E., Glover L.A., Prosser J.I. (1999). Molecular Analysis of Bacterial Community Structure and Diversity in Unimproved and Improved Upland Grass Pastures. Appl. Environ. Microbiol..

[41] Griffiths R.I., Thomson B.C., James P., Bell T., Bailey M., Whiteley A.S. (2011). The Bacterial Biogeography of British Soils. Environ. Microbiol..

[42] Lauber C.L., Hamady M., Knight R., Fierer N. (2009). Pyrosequencing-Based Assessment of Soil pH as a Predictor of Soil Bacterial Community Structure at the Continental Scale. Appl. Environ. Microbiol..

[43] Goldfarb K.C., Karaoz U., Hanson C.A., Santee C.A., Bradford M.A., Treseder K.K., Wallenstein M.D., Brodie Eoin L. (2011). Differential Growth Responses of Soil Bacterial Taxa to Carbon Substrates of Varying Chemical Recalcitrance. Front. Microbiol..

[44] Ramirez K.S., Craine J.M., Fierer N. (2010). Nitrogen Fertilization Inhibits Soil Microbial Respiration Regardless of the Form of Nitrogen Applied. Soil Biol. Biochem..

[45] Zhao C., Ni H., Zhao L., Zhou L., Borrás-Hidalgo O., Cui R. (2020). High Nitrogen Concentration Alter Microbial Community in Allium Fistulosum Rhizosphere. PLoS ONE.

[46] Wang X. (2024). Litter Quality Regulates Litter Decomposition Process in an Alpine Meadow: Based on Microbial Communities in Both Litter and Soil Layers and Their Enzymatic Stoichiometry. Ph.D. Thesis.

[47] Manici L.M., Caputo F., De Sabata D., Fornasier F. (2024). The Enzyme Patterns of Ascomycota and Basidiomycota Fungi Reveal Their Different Functions in Soil. Appl. Soil Ecol..

[48] Feng X. (2021). Effects and Mechanisms of Green Waste Compost Impacts on Nursery Soil Fertility. Ph.D. Thesis.

[49] Mora-Salguero D., Ranjard L., Morvan T., Dequiedt S., Jean-Baptiste V., Sadet-Bourgeteau S. (2025). Long-Term Effect of Repeated Application of Pig Slurry Digestate on Microbial Communities in Arable Soils. Heliyon.

[50] Aranda V., Macci C., Peruzzi E., Masciandaro G. (2015). Biochemical Activity and Chemical-Structural Properties of Soil Organic Matter after 17 Years of Amendments with Olive-Mill Pomace Co-Compost. J. Environ. Manag..

[51] Cui J., Holden N.M. (2015). The Relationship between Soil Microbial Activity and Microbial Biomass, Soil Structure and Grassland Management. Soil Tillage Res..

[52] Adetunji A.T., Lewu F.B., Mulidzi R., Ncube B. (2017). The Biological Activities of β-Glucosidase, Phosphatase and Urease as Soil Quality Indicators: A Review. J. Soil Sci. Plant Nutr..

[53] Koçak B. Importance of Urease Activity in Soil. Proceedings of the International Scientific and Vocational Studies Congress—Science and Health (BILMES SH 2020).

[54] Neemisha, Sharma S., Giri B., Kapoor R., Wu Q.-S., Varma A. (2022). Soil Enzymes and Their Role in Nutrient Cycling. Structure and Functions of Pedosphere.

[55] Zhou Y., Liu H., Wang C., Liu X., Jin M. (2025). Effects of Phosphogypsum Addition on Soil Fertility and Enzyme Activity of the Cultivated Layer in Saline-Sodic Paddy Fields. Soils.

[56] Kandeler E., Eder G. (1993). Effect of Cattle Slurry in Grassland on Microbial Biomass and on Activities of Various Enzymes. Biol. Fertil. Soils.

[57] Abubaker J., Risberg K., Pell M. (2012). Biogas Residues as Fertilisers—Effects on Wheat Growth and Soil Microbial Activities. Appl. Energy.

[58] Rahaman M.A., Zhang Q., Shi Y., Zhan X., Li G. (2021). Biogas Slurry Application Could Potentially Reduce N_2_O Emissions and Increase Crop Yield. Sci. Total Environ..

[59] Zhang S. (2022). Study on Green Production Technology of Rice in Coastal Areas Based on Application of Biogas Slurry and Soil Fertility Substrate. Master’s Thesis.

[60] Seafatullah M., Hoque M.A., Islam M.S., Islam M.M., Islam M.N. (2015). Effect of Cow Dung, Biogas Slurry and Vermicompost on Phosphorus Adsorption Behavior of Soil. J. Sci. Res..

[61] Slepetiene A., Kochiieru M., Jurgutis L., Mankeviciene A., Skersiene A., Belova O. (2022). The Effect of Anaerobic Digestate on the Soil Organic Carbon and Humified Carbon Fractions in Different Land-Use Systems in Lithuania. Land.

[62] Han C., Zhang H., Zhao L., Zhu J., Shi X., Zhang Z., Liu K., Yan D. (2025). Effects of Sorbitol-Chelated Potassium Through Foliar Spraying on Wheat Yield, Potassium Availability, and Rhizosphere Microbiome. Soils.

[63] He Y. (2022). Effects of Green Manure Incorporation on Soilnutrients, Bacterial Community Structure and Maize N and P Use Efficiency in Yellow Soil of Guizhou. Master’s Thesis.

[64] Zhang M. (2022). Effect of Magnetized Water Irrigarion on Cadmium Absorption by Sedum Alfredii. Master’s Thesis.

[65] Verma S., Pradhan S.S., Singh A., Kushuwaha M. (2024). Effect of Organic Manure on Different Soil Properties: A Review. Int. J. Plant Soil Sci..

[66] Lazcano C., Zhu-Barker X., Decock C. (2021). Effects of Organic Fertilizers on the Soil Microorganisms Responsible for N_2_O Emissions: A Review. Microorganisms.

[67] Meng X., Zeng B., Wang P., Li J., Cui R., Ren L. (2022). Food Waste Anaerobic Biogas Slurry as Fertilizer: Potential Salinization on Different Soil Layer and Effect on Rhizobacteria Community. Waste Manag..

[68] Mdlambuzi T., Tsubo M., Muchaonyerwa P. (2022). Maize (*Zea mays* L.) Production from Co-Application of Biogas Slurry with Chemical Fertilizer and Effects on Soil Quality in a Semi-Arid Region of South Africa. Commun. Soil Sci. Plant Anal..

